# Borderline ovarian tumours: management in the era of fertility-sparing surgery

**DOI:** 10.3332/ecancer.2020.1031

**Published:** 2020-05-06

**Authors:** Mattia Maramai, Fabio Barra, Mario Valenzano Menada, Sara Stigliani, Melita Moioli, Sergio Costantini, Simone Ferrero

**Affiliations:** 1 Department of Neurosciences, Rehabilitation, Ophthalmology, Genetics, Maternal and Child Health (DiNOGMI), University of Genova, Italy; 2 Academic Unit of Obstetrics and Gynaecology, IRCCS Ospedale Policlinico San Martino, Genova, Italy

**Keywords:** ovarian neoplasms, ovarian cysts, fertility preservation, salpingo-oophorectomy

## Abstract

Borderline ovarian tumours (BOTs) are ovarian neoplasms characterised by epithelial proliferation, variable nuclear atypia and no evidence of destructive stromal invasion. BOTs account for approximately 15% of all epithelial ovarian cancers. Due to the fact that the majority of BOTs occur in women under 40 years of age, their surgical management often has to consider fertility-sparing approaches. The aim of this mini-review is to discuss the state of the art of fertility-sparing surgery for BOTs with a specific focus on the extent of surgery, post-operative management and fertility.

## Introduction

Borderline ovarian tumours (BOTs) are a group of ovarian neoplasms described as ‘semimalignant disease’ for the first time by Taylor [1] in 1929. BOTs are characterised by higher epithelial proliferation and more variable nuclear atypia than benign lesions; however, they have not stromal invasion, in contrast to carcinomas [2]. The vast majority of BOTs have serous or mucinous histotypes; other rare types are clear-cell, endometrioid and Brenner (transitional-cell) tumours. Despite the lack of stromal invasion, serous BOTs may present implants on peritoneal surfaces, which in a small percentage of cases, invade underlying tissue. In the presence of invasive implants, according to the 2014 WHO (World Health Organization) Classification of Tumours of Female Reproductive Organs, BOTs should be considered as low-grade serous carcinoma [2, 3].

BOTs account for approximately 15% of all epithelial ovarian cancers, with an annual prevalence of 1.8–4.8/100,000 [4, 5]. Overall, being characterised by a less aggressive biological behaviour, BOTs have a better prognosis than malignant epithelial ovarian cancers; in fact, studies with long term follow-up describe 5-year survival rates over 90% [6–8]. Additionally, compared to malignant ovarian cancers, these tumours tend to more frequently occur in younger patients and at an earlier stage: more than a third of BOTs occur in women younger than 40 years of age, in contrast to malignant epithelial ovarian cancers, whose incidence is about 6% in the same age group. In two large series by Trillsh and du Bois, the diagnosis of BOTs was done at stage I in 78%–82% of cases [9, 10]. Several studies reported increasing in BOTs survival rates from 60’s to present, probably due to the common misclassification with carcinomas; in fact, BOTs had a first specific definition by WHO in 1973; the same studies reported an increase in their incidence in the same period as well [4]; these reports could be partly attributed to a rising awareness of the distinctive pathologic features of BOTs [2, 11].

Surgery is the primary treatment for BOTs. Similarly to the management of malignant ovarian cancers, the extent of surgery depends on stage: for stage I disease, the therapeutic approach should include a surgical staging with total hysterectomy, bilateral salpingo-oophorectomy, omentectomy, peritoneal washing and multiple biopsies; appendix should be removed in case of mucinous histology [12, 13]. In patients with evidence of advanced stage, all visible disease should be surgically removed [12, 14]. The use of minimally invasive or traditional open techniques has been evaluated in the current literature: a series of 105 patients treated by Trillsch *et al* [15] demonstrated no statistically significant differences in achieving a complete surgical cytoreduction by laparoscopy or laparotomy; moreover, the authors found an advantage in performing a complete surgical staging during primary surgery by laparotomy although these data equalised after re-staging procedures. Another series by Desfeux *et al* [16] reported no difference in recurrence rates and similar survival curves across these surgical approaches. For this reason, minimally invasive surgery is considered feasible and safe in the management of patients affected by BOTs. Casarin *et al* [17] analysed the impact of adnexal size on the risk of surgical spillage: in their retrospective analysis, adnexa larger than 10 cm in maximum diameter were associated with a 4-fold risk of surgical spillage with laparoscopic approach (54.5% versus 12.1%) compared to open surgery. In the sub-analysis of patients treated by laparoscopy, they found a positive correlation between the adnexa size and surgical conversion rates (*p* = 0.003), need for mini laparotomy for retrieving the specimens (*p* = 0.006) and global operative time (*p* < 0.001). Nevertheless, laparoscopy obtained less intraoperative blood loss (*p* = 0.007) and shorter operative time (*p* < 0.001).

The increasing use of robotic surgery in gynaecology was focalised also on the management of early-stage ovarian cancer and BOTs. In fact, the robotic-assisted approach is useful and safe in patients with presumed early stage disease [18]. In a retrospective analysis of 39 patients treated by minimally invasive surgery for presumed early-stage ovarian cancer, Bellia *et al* [19] showed no significant difference between laparoscopic versus robotic management in terms of operating time, needs for blood transfusion, intra- and post-operative complications and number of lymph nodes detected. Similar results were found by Nehzat *et al* [20] in a retrospective analysis of 63 patients with a presumed stage I ovarian cancer who underwent minimally invasive surgery. No difference was found in terms of estimated blood loss, length of stay and operative time.

Overall, the choice of surgical approach for BOTs should consider size of ovarian masses, presence and localisation of peritoneal implants, presence of bulky nodes, surgeon’s skills and patient’s individual characteristics [21, 22]. Another topic for debate on surgical management of early-stage BOTs is represented by the need for systematic lymphadenectomy: in a retrospective analysis by Matsuo *et al* [23], no difference was found in survival rates in patient undergoing lymphadenectomy. Despite extreme variability among the various series, up to 40%–50% of lymph node involvement has been reported in BOTs [24, 25]. However, the presence of positive lymph nodes does not seem to affect global recurrence and patient’s overall survival [23, 25]. For this reason, the vast majority of investigators do not recommend routine pelvic and para-aortic lymphadenectomy for managing these tumours; otherwise, the removal of bulky suspicious nodes is suggested [24, 26]. The role of intra-operative frozen section (FS) during surgical management of BOTs is under debate: a retrospective analysis by Ureyen *et al* [27] reported a significative difference in concordance between FS and definitive diagnosis in serous and mucinous histologies (92% versus 62%); a wide decrease in concordance were found also in ovarian masses larger than 100 mm (90% versus 68%). Another paper from a tertiary referral centre highlighted that in older patients, under-diagnosis of borderline histology after FS rises from 33.3% to 53.3% [28]. In a retrospective study Shah *et al* [29] analysed concordance between FS diagnosis and final pathologic diagnosis of BOTs when performed in an academic hospital with a gynaecologic pathologist, an academic hospital with a nongynaecological pathologist and a community hospital with a nongynaecological pathologist; no significative differences were shown regardless of hospital type [29]. Globally considering the evidence from the literature, the use of FS in suspicious ovarian masses appears to reliably distinguish benign from malignant lesions. However, as reported above, its use in BOTs needs more caution, especially in cases of mucinous histology, large masses and older patients [30]. In cases of incomplete surgical staging at primary surgery, re-intervention for achieving complete staging does not likely affect survival rates related to BOTs [31].

Because many patients are still fertile at the time of diagnosis, not having completed the desire of childbearing, fertility-sparing surgery is considered as a relevant option in the management of BOTs. In many series published, the overall survival for patients undergoing a fertility-sparing surgery is close to 100% [10, 32–34].

## Fertility-sparing approach

In woman with unilateral/bilateral ovarian involvement, fertility-sparing options include surgical procedures, such as unilateral cystectomy (US), unilateral salpingo-oophorectomy (USO), bilateral cystectomy (BC) or unilateral salpingo-oophorectomy plus contra-lateral cystectomy (USO+CC). In a French multi-centre study, including 313 patients with stage I BOTs, the recurrence rates after cystectomy, USO or BSO were 30.3%, 11% and 1.7%, respectively [35]. In a retrospective series, De Iaco *et al* [36] analysed the surgical outcome of 168 patient with affected by BOTs treated with conservative surgery between 1985 and 2006: 35 underwent US, 50 underwent USO and 83 radical surgery; recurrence rate was 34.3% in US group, 20% in USO group and 6% in radical surgery group; moreover, patients who experienced tumour relapse did not die of this disease [36]. The authors definitively suggested cystectomy as a suitable option in younger patients or women with bilateral BOT or previous history of unilateral adnexectomy, after appropriate counselling about the risk of local relapse. Vasconcelos *et al* [37] published a meta-analysis on studies, including 5,105 women, 2,725 of who underwent conservative surgery for BOTs. Among patients undergoing US, BC, USO and USO+CC, the pooled recurrence estimates were, respectively, 25.3%, 25.6%, 12.5%, and 26.1%. In sub-analysis on patients with unilateral BOTs, USO performed significantly better than US with an OR for recurrence reduction of 2.2 (95% CI = 0.793–2.841, *p* < 0.0001). Overall, in cases of bilateral BOTs, USO+CC did not obtain an advantage compared to BC in terms of recurrence (26.1% versus 25.6); therefore, less destructive approaches in this setting may be considered in order to preserve patients’ fertility. Although no differences were found between conservative and radical surgery in terms of overall survival, the authors concluded that the low mortality rate precludes pooling estimation for death in relation to the different types of fertility sparing surgery; moreover, the short-term follow-up times tend to limit the interpretation of survival analysis. In this meta-analysis, the only prospective randomised controlled trial was an Italian paper published by Palomba *et al* [38], which compared BC and USO+CC, obtaining a significant shorter time to first recurrence in the BC group (*p* < 0.01); however, performing a regression analysis, the difference did not reach a statistical significance (*p* = 0.14); additionally, disease recurrence (1.23 [95% CI, 0.62–3.17; *p* = 0.41]) was not different between these groups.

Obtaining a biopsy from a normal appearing contra-lateral ovary is not recommended in patients undergoing surgical management for BOTs because the risk of under-diagnosis of an occult malignancy tends to be very low [33]. Furthermore, this procedure may cause adhesions that may impact negatively on fertility in 10%–20% of the cases; in fact, in cases of extensive biopsies, the risk of favouring the onset of iatrogenic menopause has been described [39].

The management of relapse of BOTs mostly depends on tumour location and histotype. In case of a relapse in remnant ovarian tissue, a second conservative surgery could represent a suitable treatment option in patients desiring to preserve fertility [40]; otherwise, in case of extra-ovarian relapse the surgical approach should be based on a complete cytoreductive surgery, associated with platinum/taxane-based chemotherapy in case of progression to low-grade serous carcinoma [14, 41]. Trans-vaginal ultrasonography seems to be the most effective technique in follow-up of patients treated conservatively for BOTs [42].

## Pregnancy rates and infertility after fertility-sparing approach

It is difficult to exactly assess the impact of fertility-sparing treatments for BOTs on ovarian function and fertility. It has been reported that approximately 81% of women retain normal menstrual cycles after conservative surgery for BOTs [43]. As in benign diseases like endometriosis, the aim of fertility preservation after fertility-sparing surgery on the ovaries is debated [44]. In a meta-analysis by Raffi *et al* [45] the serum anti-Müllerian hormone (AMH) decreased by 38% after US or BC for ovarian endometriomas. Chang *et al* [46] also found a decrease in AMH serum levels after laparoscopic ovarian cystectomy; However, they observed a restore in AMH level after 3 months of follow-up, with no statistically difference with preoperative AMH levels. The same result was showed by Vignali *et al* [47] among patients underwent US or BC for ovarian endometriomas: no differences was shown between preoperative and 12-months postoperative AMH levels (*p* > 0.05); moreover, no differences in antral follicle count (AFC) were shown [48].

Pregnancy rates in women attempting to conceive after fertility-sparing surgery are very heterogeneous [49]. In the current literature, the main published series report pregnancy rates after treatment for early stage BOTs ranging from 30% to 80% [50, 51]. A retrospective analysis by Delle Marchette *et al* [52] did not show differences in terms of pregnancy rates with regard to surgical approach (open versus laparoscopy) or type of surgery (UC versus USO). However, the need for additional surgical procedures reduced the probability of getting pregnant by about 40% [52].

Another controversial topic is the need to use assisted reproductive techniques for improving fertility outcomes. In selected patients, induction of ovulation and *in vitro* fertilisation may be required after fertility-sparing surgery in order to enhance the chance of conceiving. Potential associations between ovulation-inductor drugs and BOTs have been proposed by several authors: in 1994, Rossing *et al* [53] highlighted the relation between the use of clomiphene and the increased risk to develop BOTs or even invasive ovarian cancer. Subsequent studies reduced the emphasis on this association, which at the moment is characterised by low evidence [54–56]. A meta-analysis by Siristatidis *et al* [57] showed a statistically significant correlation between controlled ovarian hyper-stimulation and ovarian cancer (RR = 1.50, 95% CI: 1.17–1.9); however, when the analysis was based only on infertile women, no significant associations were noted (RR = 1.26, 95% CI: 0.62–2.55); with regard to BOTs, no conclusions have been drawn due to not negligible study limitations, such as short follow-up period, low statistical power and absence of control groups [41].

As general rule, fertility counselling should be mandatory in the management of BOTs among women aiming to spare fertility. Patients with diagnosis of BOTs should be referred to an oncofertility centre before surgery in order to assess their reproductive status and to plan subsequent operative management [58, 59].

## Surveillance after fertility-sparing approach

Currently, there is no universally accepted standard-of-care regarding follow-up after surgery for BOTs. In a cohort of 39 women, Uzan *et al* [60] reported that the vast majority of non-invasive relapse of BOTs were diagnosed by ultrasound (16/23; 69%); CA125 elevation occurred in case of progression to invasive ovarian cancers (6/13; 46%) [60]. Same evidence was found by Zanetta *et al* [42] in a cohort of 28 women experiencing a relapse of BOTs. Frequency of post-operative visits and chose of exams are mainly dependent to the institutional or physician expertise; the performance of clinical examination, ultrasonography and serum tumour markers is widely accepted. Staging Classifications and Clinical Practice Guidelines for Gynaecological Cancers, edited by the Fédération Internationale de Gynécologie et d’Obstétrique in 2000 suggest that a control visit should be carried out every three months during the first year after surgery for invasive ovarian cancer, with an increase to every four to six months and every year after the fifth year. For BOTs, this frequency can be annual [61]. As in the case of fertility-sparing surgery, the majority of relapses occur in the remnant ovary, a routinely ovarian scan by ultrasound in the hands of a skilled operator should be done; computed tomography or magnetic resonance are second-line options, although their use appears pivotal in the case of elevation of serum CA125. In a multicentre prospective study, Fisherova *et al* [62] analysed the outcomes of 20 patients with a previous diagnosis of BOT and a recurrent ovarian mass: in this population a subjective transvaginal ultrasonographic assessment of the recurrent ovarian mass by a skilled operator obtained a sensitivity of 94% with a false-positive rate of 33%.

## Disease relapse after fertility-sparing approach

The main clinical factors associated with disease relapse are advanced age at diagnosis, preoperative elevation of serum levels of CA125, presence of invasive implants and micropapillary histology [63].

Rates of relapse described after fertility sparing surgery for BOTs greatly differ in the current literature. The evidence shows that serous BOTs recurred more frequently than mucinous BOTs, despite progressing to an invasive carcinoma only in a smaller percentage of cases. In a retrospective analysis by Uzan *et al*. [64], 191 of 254 women (75.2%) underwent conservative management for BOTs; the authors found 43 cases of recurrence (26 serous BOT, 17 mucinous BOT; *p* = 0.01); among the women with initial diagnosis of serous BOT, only 3 (11.5%) developed an invasive carcinoma; in contrast, among those with initial diagnosis of mucinous BOT, invasive carcinoma occurred in 9 women (52.9%).

Recurrence of serous BOT in residual ovary almost always has a non-invasive histology; for this reason, it should be considered as a new primary BOT and could be potentially treated by a second fertility-sparing surgery; conversely, the vast majority of invasive recurrences are characterised by extra-ovarian involvement [14,65]. For mucinous BOT the relapse on residual ovary is related to disease persistence after an incomplete primary surgery, while extra-ovarian relapse is associated with higher risk of invasive histology and poor prognosis [66–67].

In a prospective observational study, Franchi *et al* [68] used transvaginal ultrasonography to assess the growth of recurrent ovarian cyst. They followed up patients with previous conservatively treated BOT and new ovarian cyst until the clinical setting recommended proceeding with surgery (no evidence of metastasis, no ascites, maximum diameter of the suspected recurrent lesion <40 mm, presence of ‘ovarian crescent sign’ and negative tumor marker). The median time prior to surgery was 9.8 months; the final histological report confirmed the previous histotype in all the patients [68].

## Surgery completion after fertility-sparing approach

Recurrence rates in fertility-sparing surgery for BOTs are higher than after radical surgery; however, after the completion of desire of conception the surgical second look with removal of uterus and contra-lateral ovary remains debated. In fact, the published data does not report differences in terms of survival rates after completion of surgery for BOTs. For serous BOTs many authors suggest expectant management and performance of radical surgery only in cases of disease recurrence [13, 69]. For mucinous BOTs several authors suggest the completion of surgery, in fact, many mucinous tumours relapsed as invasive ovarian cancers [70]. In these young patients, psychological impact of disease should not be neglected; thus, an accurate and comprehensive preoperative fertility counselling has to be done [71].

## Conclusion

Fertility sparing surgery for BOTs is feasible and does not seem to negatively influence patients’ long-term survival, although higher disease recurrence rates have been reported. The extent of surgery should be individualised based on patient characteristics, tumour stage and histology. Women with a diagnosis of BOT should be referred to an oncofertility centre prior to performing fertility-sparing surgery in order to assess reproductive status and to plan future post treatment pregnancies. In these patients, a routine follow-up evaluation should be done, including clinical examination, ultrasound and dosage of serum tumour markers. Surgical management of relapse depends on disease localisation and histology.

## Conflicts of interest

The authors have not conflict of interest to declare.

## Authors’ contributions

MMA: literature review, manuscript writing
FB: manuscript revision
MVM: literature review
SS: data analysis
MMO: data analysis
SF: manuscript revision
SC: supervision

## Funding statement

This paper has not been funded.
